# Neutrophils Dampen Adaptive Immunity in Brucellosis

**DOI:** 10.1128/IAI.00118-19

**Published:** 2019-04-23

**Authors:** Ricardo Mora-Cartín, Cristina Gutiérrez-Jiménez, Alejandro Alfaro-Alarcón, Esteban Chaves-Olarte, Carlos Chacón-Díaz, Elías Barquero-Calvo, Edgardo Moreno

**Affiliations:** a Programa de Investigación en Enfermedades Tropicales (PIET), Escuela de Medicina Veterinaria, Universidad Nacional, Heredia, Costa Rica; b Departamento de Patología, Escuela de Medicina Veterinaria, Universidad Nacional, Heredia, Costa Rica; c Centro de Investigación en Enfermedades Tropicales (CIET), Facultad de Microbiología, Universidad de Costa Rica, San José, San Pedro, Costa Rica; University of California San Diego School of Medicine

**Keywords:** *Brucella*, *Brucella abortus*, interferon gamma, adaptive immunity, brucellosis, native immunity, neutralizing antibodies, neutrophils

## Abstract

*Brucella* organisms are intracellular stealth pathogens of animals and humans. The bacteria overcome the assault of innate immunity at early stages of an infection. Removal of polymorphonuclear neutrophils (PMNs) at the onset of adaptive immunity against Brucella abortus favored bacterial elimination in mice. This was associated with higher levels of interferon gamma (IFN-γ) and a higher proportion of cells expressing interleukin 6 (IL-6) and inducible nitric oxide synthase (iNOS), compatible with M1 macrophages, in PMN-depleted B. abortus-infected (PMNd-*Br*) mice. At later times in the acute infection phase, the amounts of IFN-γ fell while IL-6, IL-10, and IL-12 became the predominant cytokines in PMNd-*Br* mice. IL-4, IL-1β, and tumor necrosis factor alpha (TNF-α) remained at background levels at all times of the infection. Depletion of PMNs at the acute stages of infection promoted the premature resolution of spleen inflammation. The efficient removal of bacteria in the PMNd-*Br* mice was not due to an increase of antibodies, since the immunoglobulin isotype responses to *Brucella* antigens were dampened. Anti-*Brucella* antibodies abrogated the production of IL-6, IL-10, and IL-12 but did not affect the levels of IFN-γ at later stages of infection in PMNd-*Br* mice. These results demonstrate that PMNs have an active role in modulating the course of B. abortus infection after the adaptive immune response has already developed.

## Abstract (precis)

*Brucella* organisms are intracellular stealth pathogens of animals and humans. The bacteria overcome the assault of innate immunity at early stages of an infection.

## INTRODUCTION

Polymorphonuclear neutrophils (PMNs) are essential elements of innate immunity and the first line of defense against microbial invaders. These cells phagocytize and destroy bacteria, release cytokines, and activate elements of the innate immune response (1). However, PMNs also modulate components of adaptive immunity, a phenomenon that has gained considerable attention in recent years (2, 3).

Neutropenic murine models have been used to dissect the roles of PMNs during innate and adaptive immune responses against microbial infections. The selective depletion of PMNs by means of antibodies is the most common and widespread model (4–8). A second model includes a mutant mouse strain named Genista, which is devoid of mature PMNs (4, 9–11). Both models have advantages and drawbacks, though they generally display good correlation and render similar results (4, 11). Neutropenia in the anti-PMN depletion model is transient and cannot be maintained beyond 1 week. Still, the advantage of this model is that the neutropenic condition can be induced at any stage of an infection (12–14).

We have used both the Genista and anti-PMN models to explore the role of PMNs and innate immune response during the onset of Brucella abortus infection (4, 15). *Brucella* organisms are intracellular stealth pathogens of animals and humans that avoid the activation of innate immunity, remaining in several tissues for protracted periods (15–17). B. abortus readily invades PMNs, resisting the killing action of these leukocytes (15, 18–22). This correlates with the resistance and modification of the bacterial cell envelope components, which barely promote the generation of reactive oxygen species and proinflammatory cytokines in the infected PMNs (15, 19). In addition, through its lipopolysaccharide (*Br*-LPS), B. abortus mediates in a nonphlogistic manner the premature cell death of PMNs and induces the expression of “eat me” signals on these cells (19, 21). The absence of PMNs at the onset of B. abortus infection stimulates the recruitment of monocytes/dendritic cells, favors the activation of B and T lymphocytes, and promotes the production of Th1 cytokines (4).

The course of human brucellosis parallels that observed in mice (16, 23). In the mouse model, brucellosis is divided into four phases according to the bacterial colonization of the target organs, the pathological signs, and the profile of the immune response (17, 23). The first phase corresponds to the onset of infection (also known as the incubation stage), which typically lasts 2 to 3 days. During this phase, the production of proinflammatory cytokines and the activation of innate immunity are negligible (4). The acute phase follows, lasting 2 to 3 weeks. Active bacterial replication and high levels of Th1 cytokines characterize this phase (23, 24). Then, the chronic steady phase, lasting from 8 to 11 weeks, corresponds to the plateau of the infection. Finally, the chronic declining phase is characterized by the gradual elimination of bacteria. This phase may last months or even years (23, 24). During the acute and chronic phases, large amounts of anti-*Br*-LPS antibodies are produced (25). At these stages, the bone marrow (BM) is colonized by *Brucella* organisms, maintaining for protracted periods high bacterial loads within BM PMNs and, to a minor extent, in monocytes and stem cells (17). This is significant, since PMNs in other target organs, such as the spleen, do not harbor *Brucella* (26).

Here, we describe how PMNs modulate adaptive immunity in the initial stages of acute murine brucellosis. The results presented here reinforce our previous hypothesis (4) and give new insights into the role that PMNs have in shaping the immune response during brucellosis.

## RESULTS

### The absence of PMNs enhances the removal of B. abortus in mice.

We have shown that the absence of PMNs at the onset of B. abortus infection enhances bacterial removal after several days (4). Following this, we explored whether the absence of PMNs has any influence at the onset of adaptive immunity, once Th1 cytokines and specific antibodies have developed (21). For this, the protocols described in Fig. S1A and B in the supplemental material were followed.

**FIG 1:**
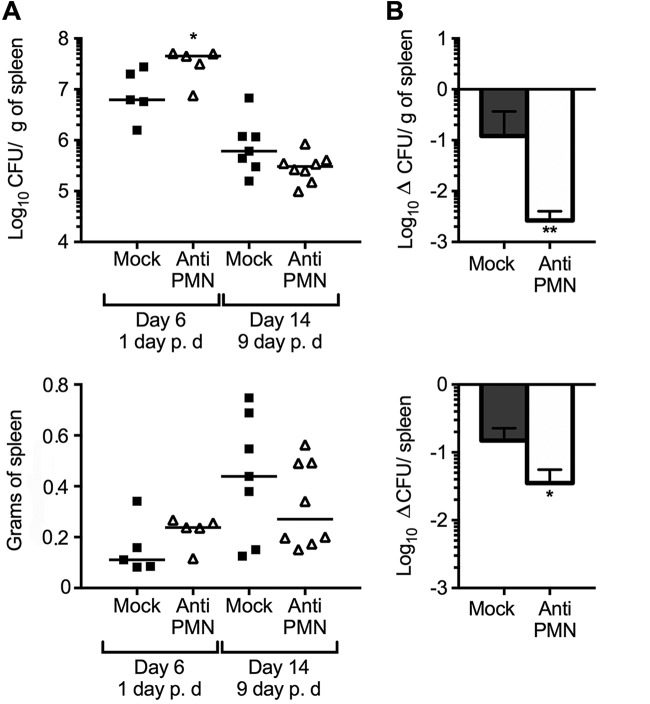
PMN depletion at the onset of adaptive immunity promotes *Brucella* removal. C57BL/6 mice were i.p. infected with 0.1 ml of PBS containing 10^6^ CFU of B. abortus 2308W. After 5 days of infection, one group of mice was depleted of PMNs by means of i.p. injection of RB6-8C5 anti-PMN. (A) At the indicated times, the numbers of CFU per spleen and spleen weights were determined. Each symbol represents one animal, and the lines represent the medians for each group. p. d, postdepletion. (B) Rates of change in CFU per spleen (Δ CFU/spleen) and CFU per spleen weight (Δ CFU/g of spleen) were calculated over time using the following equations: Δ CFU/spleen = mean CFU 14 days/CFU 6 day ± standard deviation (SD) and Δ CFU/g of spleen = mean CFU/g of spleen 14 days/6 days ± SD. The error bars represent standard deviations. *, *P* < 0.05, and **, *P* < 0.01, in relation to the mock-treated controls.

**FIG 2:**
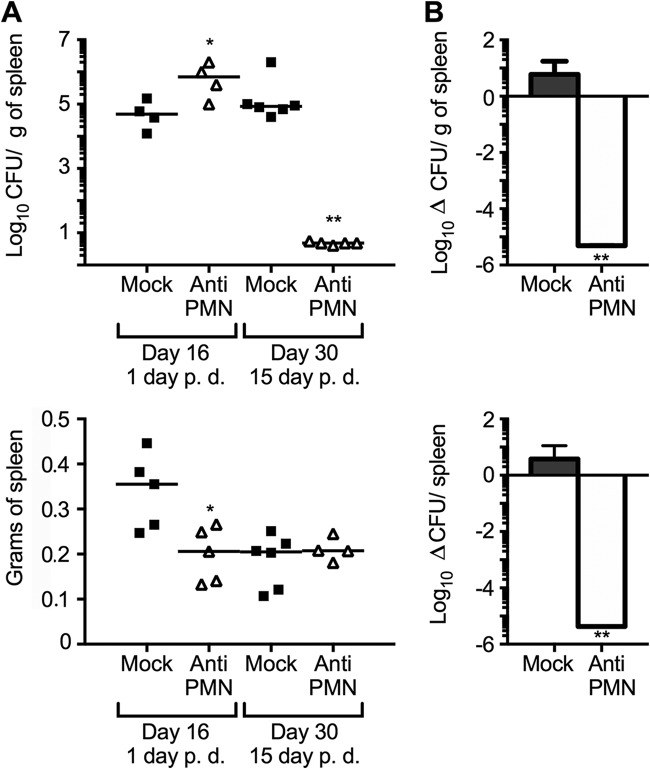
PMN depletion during the acute infection period promotes *Brucella* removal. C57BL/6 mice were i.p. infected with 0.1 ml of PBS containing 10^6^ CFU of B. abortus 2308W. After 15 days of infection, one group of mice was depleted of PMNs by means of i.p. injection of RB6-8C5 anti-PMN. (A) At the indicated times, the numbers of CFU per spleen and spleen weights were determined. Each symbol represents one animal, and the lines represent the medians for each group. (B) Rates of change in CFU per spleen (Δ CFU/spleen) and CFU per spleen weight (Δ CFU/g of spleen) were calculated over time using the following equations: Δ CFU/spleen = mean CFU 30 days/CFU 6 day ± SD and Δ CFU/g of spleen = mean CFU/g of spleen 30 days/16 days ± SD. The error bars represent standard deviations. **, *P* < 0.01 in relation to the mock-treated controls.

After the sixth day of infection (1 day after PMN depletion), we observed an initial increase of bacterial loads in the spleens of PMNd-*Br* mice (Fig. 1A). This outcome agreed with our previous results (4). After 14 days of B. abortus infection (9 days post-PMN depletion), the numbers of CFU in the spleens of PMNd-*Br* mice reached values similar to those of the non-PMN-depleted controls (Fig. 1A); however, PMNd-*Br* mice showed more efficient bacterial removal (Fig. 1B). This phenomenon was more conspicuous after 30 days of infection (15 days of PMN depletion) (Fig. 2).

**FIG 3:**
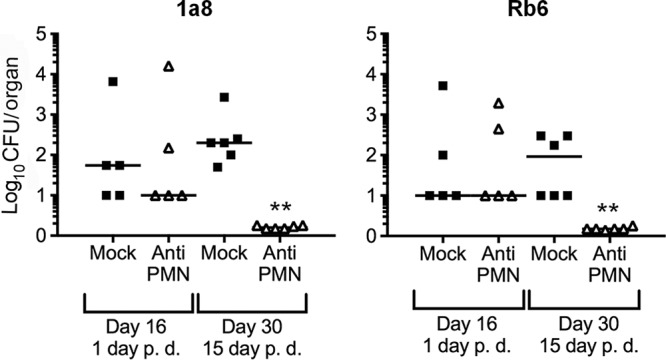
Late PMN depletion diminishes bacterial loads in BM. C57BL/6 mice were i.p. infected with 0.1 ml of PBS containing 10^6^ CFU of B. abortus 2308W, and at 15 days postinfection, one group of mice was depleted of PMNs by means of i.p. injection of 1A8 or RB6-8C5 anti-PMN. At the indicated times, the number of CFU per BM was determined. Each symbol represents one animal. **, *P* < 0.01 in relation to the mock-treated controls.

RB6-8C5 antibody partially depletes a subpopulation of monocytes (27) (see Table S1 in the supplemental material). Therefore, we repeated the experiment using the anti-PMN antibody from the 1A8 clone, which is supposed to be highly specific for murine PMNs (27). Similar results using this antibody were observed (see Fig. S2 in the supplemental material). However, the elimination of bacteria was more evident in BM, regardless of the antibody used to deplete PMNs (Fig. 3). This was striking, since during the chronic stages, the presence of *Brucella* organisms in the BM is marked (17), and in contrast to other tissues, BM retains a proportion of PMNs after depletion of these cells (see Table S1).

It is worth mentioning that the 1A8 antibody has several drawbacks in comparison to the RB6-8C5 antibody. To achieve significant PMN depletion, very high doses of 1A8 antibody (500 μg/mouse) were required. In spite of this, depletion seldom reached more than 95% of blood PMNs (see Table S1), and neutropenia was not as steadily maintained as with the RB6-8C5 antibody. Similar results have been reported by other authors (27). Regardless of this, the overall elimination of bacteria was more efficient in the PMNd-*Br* mice than in the infected controls.

### B. abortus infection enhances cytokine production in neutropenic mice.

**FIG 4:**
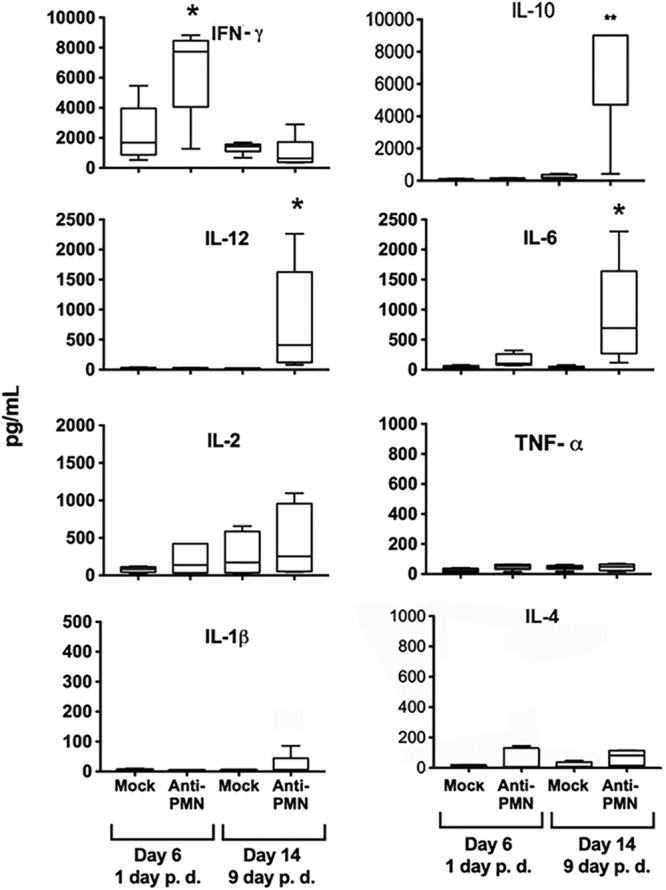
PMN depletion after B. abortus infection increases the levels of cytokines. C57BL/6 mice were infected by the i.p. route with 0.1 ml of PBS containing 10^6^ CFU of B. abortus 2308W. After 5 days of infection, one group of mice was depleted of PMNs by means of i.p. injection of RB6-8C5 anti-PMN. The levels of various cytokines were determined by ELISA in the sera of all the mice at 6 and 14 days postinfection (1 and 9 days postdepletion, respectively). Bars represent the value distribution, while the median values are indicated by the horizontal lines within bars. Whiskers above and below the bars represent the error values. *, *P* < 0.05, and **, *P* < 0.01, in relation to the mock-treated controls.

At the onset of B. abortus infection, the levels of proinflammatory cytokines are negligible. This agrees with the stealth strategy of *Brucella* (15). However, once an infection has been established (after 5 days), there is an increase in production of interferon gamma (IFN-γ), the most relevant cytokine for mounting an efficient immune response against *Brucella* sp. infections (28, 29). As expected, after 6 days of infection, the levels of IFN-γ were already high in the mock-treated control mice (Fig. 4). Still, the amounts of IFN-γ doubled in PMNd-*Br* mice (1 day of PMN depletion), with negligible or low production of other cytokines (Fig. 4). After 14 days of infection (9 days of PMN depletion), the levels of IFN-γ decreased, but the regulatory interleukin 10 (IL-10) and other cytokines, such as IL-12 and IL-6, significantly increased (Fig. 4). Similar results for the levels of IFN-γ were observed using the 1A8 antibody for PMN depletion (see Fig. S3 in the supplemental material).

**FIG 5:**
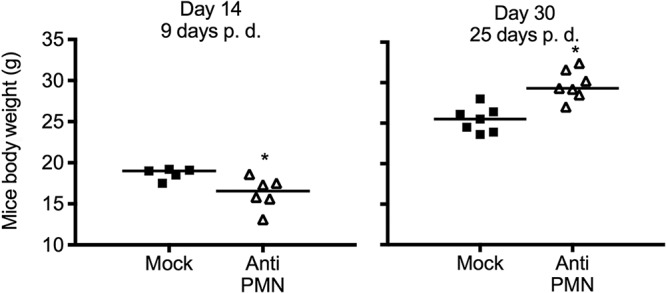
PMN depletion after B. abortus infection induces reduction in body weight. C57BL/6 mice were i.p. infected with 0.1 ml of PBS containing 10^6^ CFU of B. abortus 2308W and depleted of PMNs with RB6-8C5 antibodies 5 days postinfection. Mouse body weights at day 14 postinfection (9 days postdepletion) and day 30 postinfection (25 days postdepletion) are shown. Each symbol represents one animal. *, *P* < 0.05 in relation to the mock-treated controls.

Unexpectedly, the day after PMN depletion (6 days of B. abortus infection), the PMNd-*Br* mice showed clinical symptoms, such as lethargy, piloerection, anorexia, and general malaise. Weight loss of the PMNd-*Br* mice was evident after 14 days of infection (9 days of PMN depletion) (Fig. 5). However, 30 days after infection (25 days of PMN depletion), the weight of the PMNd-*Br* mice increased in comparison to the mock-treated controls, suggesting health improvement due to better bacterial removal (Fig. 5). Similar results were obtained with the 1A8 antibody.

### The absence of PMNs promotes the premature resolution of spleen inflammation in infected mice.

**FIG 6:**
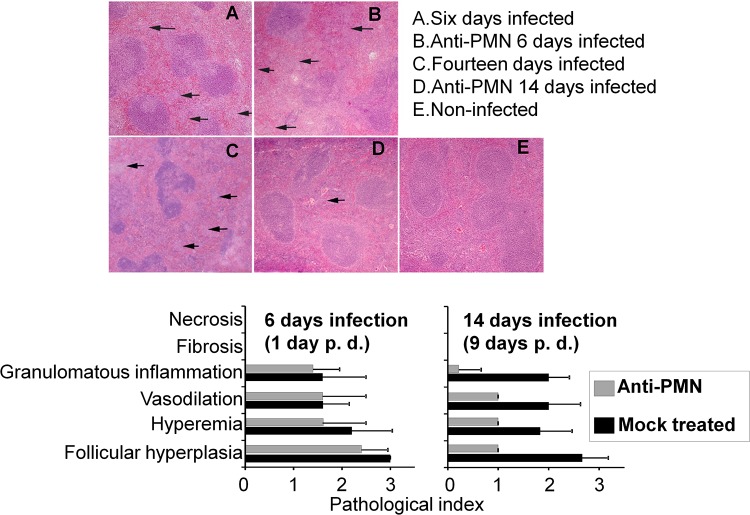
PMN depletion during the immune response favors the premature resolution of inflammation. (Top) Spleens from B. abortus 2308W-infected C57BL/6 mice were processed for histological examination and stained with hematoxylin and eosin, and the pathological parameters were observed under a microscope (magnification, ×10). The arrows indicate the presence of granulomas. (Bottom) Semiquantitative estimation of spleen inflammation by evaluating the pathological index. All values in the lower right panel at 14 days of infection (9 days after PMN depletion) were significant (*P* < 0.01 in relation to the mock-treated controls). The error bars represent standard deviations.

The removal of PMNs at the onset of B. abortus infection induces premature granulomatous inflammation and follicular hyperplasia of the spleen characterized by augmented infiltration of epithelioid histiocytes (4). In contrast, the removal of PMNs after the immune response has been established induces a different pathological effect in infected mice (Fig. 6). As expected, after 6 days of infection (1 day of PMN depletion), PMNd-*Br* mice showed no significant differences in spleen inflammation (Fig. 6A and B). However, the absence of PMNs at the acute stages of adaptive immunity favored the fast resolution of spleen inflammation (Fig. 6). Indeed, after 14 days of infection (9 days of PMN depletion), PMNd-*Br* mice showed lower numbers of granulomas, reduced vasodilation, lower follicular hyperplasia, and less hyperemia (Fig. 6C and D) than the spleens of the mock-treated controls (Fig. 6E). As shown previously (4), the depletion of PMNs alone did not induce pathological alterations in the target organs of noninfected mice.

### Neutropenic mice show lower antibody responses against B. abortus antigens.

**FIG 7:**
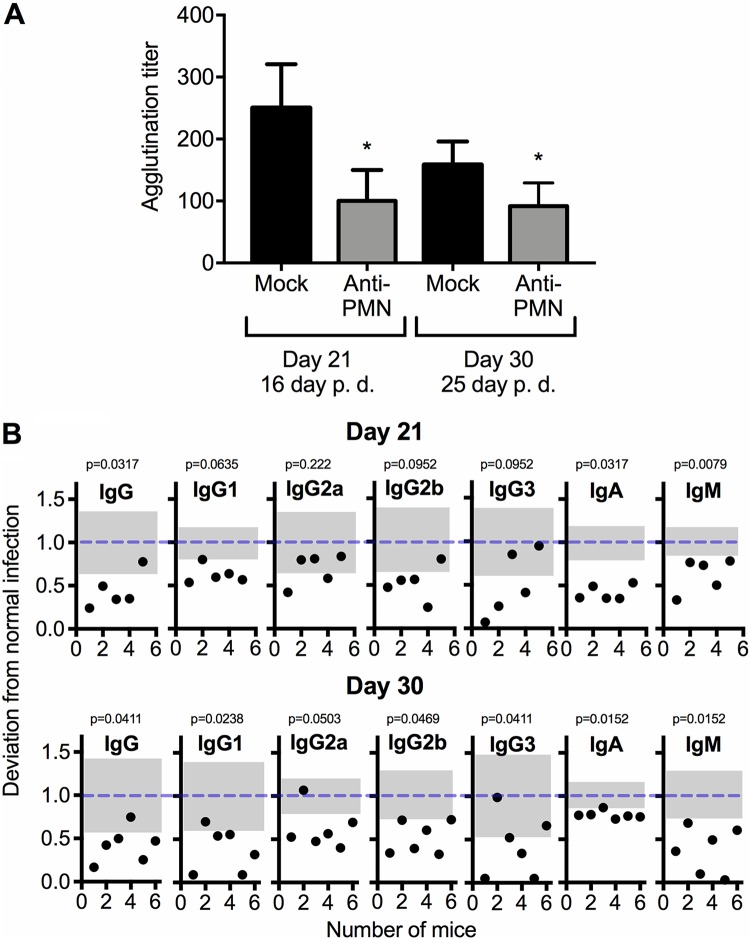
The specific antibody response is depressed in PMN-depleted mice. C57BL/6 mice were i.p. infected with 0.1 ml of PBS containing 10^6^ CFU of B. abortus 2308W, and at 5 days of infection, one group of mice was depleted of PMNs by means of RB6-8C5 anti-PMN i.p. injection. (A) Agglutination titers against *Brucella* cells. (B) Isotype antibody responses against *Br*-LPS. Each black dot represents one animal. The dashed lines show the average normalized value of the mock-treated controls, and the gray areas represent the standard deviations of the mock-treated controls. The cutoff and range values of ELISA optical densities are provided in Materials and Methods. *, *P* < 0.05 in relation to the mock-treated controls.

It has been demonstrated that IFN-γ influences the immunoglobulin isotypes against *Brucella* antigens (30). Therefore, we investigated if the reduced bacterial loads in the neutropenic mice could be due to higher antibody titers or to an increase of specific antibody isotypes against *Br*-LPS, the most relevant antigen in brucellosis (16). In comparison to the mock-treated controls, the PMNd-*Br* mice displayed lower antibody agglutination titers after 21 and 30 days of infection (16 and 25 days of PMN depletion, respectively) (Fig. 7A). These titers correlated with the generally smaller amounts of the immunoglobulin isotypes against *Br*-LPS at both times and was more evident after 30 days of infection (25 days of PMN depletion) (Fig. 7B). A similar trend was recorded after 30 days of infection (25 days of PMN depletion) when the 1A8 antibody was used for PMN depletion. However, it was less conspicuous than that observed with the RB6-8C5 antibody, and a significant increase of IgG3 production was observed (see Fig. S4 in the supplemental material).

### The absence of PMNs promotes M1 macrophage polarization.

**FIG 8:**
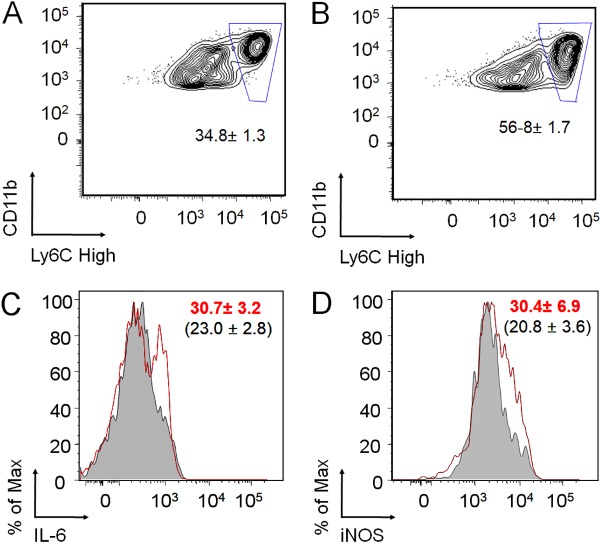
Absence of PMNs promotes M1 macrophage polarization. Lymph node leukocytes of C57BL/6 mice were analyzed by flow cytometry for CD11b, Ly6C, IL-6, and iNOS markers. (A) Lymph node leukocytes of infected mice sorted by CD11b^+^/Ly6^Hi^. (B) Lymph node leukocytes from PMN-depleted infected mice sorted by CD11b^+^/Ly6^Hi^. (C) Ly6C^+^ and Ly6C^Hi^ cells from lymph nodes were analyzed in the presence of intracellular IL-6 by flow cytometry. (D) Ly6C^+^ and Ly6C^Hi^ cells from lymph nodes were analyzed for the presence of intracellular iNOS by flow cytometry. The gray areas and the numbers within parentheses correspond to B. abortus-infected mice. The red lines marking areas and the numbers in red correspond to PMN-depleted infected mice.

*Brucella* organisms manipulate the peroxisome proliferator-activated receptor gamma (PPARγ) pathway to avoid M1 macrophage polarization and benefit from a nutrient-rich environment of alternatively activated M2 macrophages (31). Since polarization towards M1 macrophages is promoted by IFN-γ, we explored the proportion of M1 cells in the PMNd-*Br* mice. As shown in Fig. 8A and B, the relative amounts of lymph node Ly6C^+^/Ly6C^Hi^ cells were enhanced in the PMNd-*Br* mice after 9 days of infection. When Ly6C^+^/Ly6C^Hi^ cells were analyzed for intracellular IL-6 and inducible nitric oxide synthase (iNOS) (markers for M1 macrophages), the proportion of macrophages displaying these markers was higher in the PMNd-*Br* mice (Fig. 8C and D).

### Anti-*Brucella* antibodies abrogate IFN-γ production at the onset, but not at later times, of infection.

**FIG 9:**
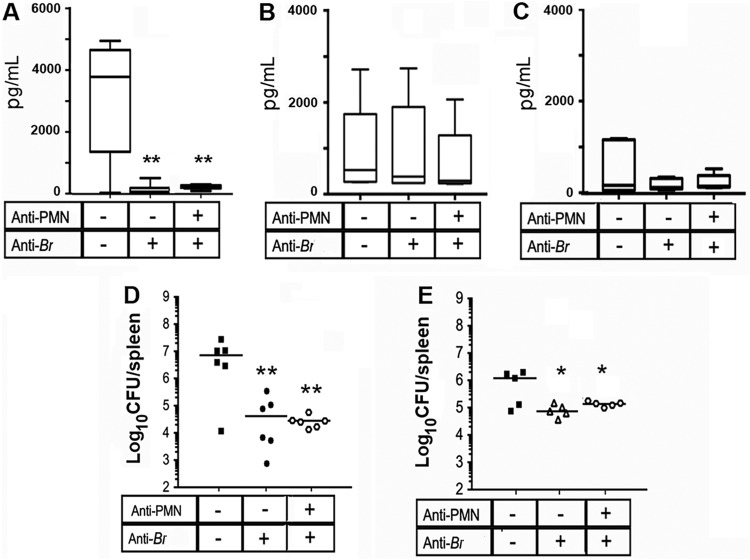
Anti-*Brucella* antibodies dampen IFN-γ production at the onset, but not at later times, of infection. (A) C57BL/6 mice were i.p. injected (+) or not (−) either with immune mouse sera against *Brucella* or with a mixture of immune mouse sera against *Brucella* and anti-PMN (RB6-8C5) 1 day before infection and 2 days after infection, and IFN-γ was measured in the sera of the mice by ELISA after 5 days of infection (see Fig. S1C). (B) Mice were i.p. injected (+) or not (−) either with immune mouse sera against *Brucella* or with a mixture of immune mouse sera against *Brucella* and anti-PMN 5 days after infection, and IFN-γ was measured after 6 days of infection (see Fig. S1D). (C) Mice were i.p. injected (+) or not (−) either with immune mouse sera against-*Brucella* or with a mixture of immune mouse sera against *Brucella* and anti-PMN 6, 9, and 12 days after infection, and IFN-γ was measured after 14 days of infection (see Fig. S1E). (D) Bacterial counts corresponding to the experiment shown in panel A. (E) Bacterial counts corresponding to the experiment shown in panel C. (A to C) Median values are indicated by the horizontal lines within bars. (D and E) Each symbol represents one animal, and the lines represent the medians for each group. *, *P* < 0.05, and **, *P* < 0.01, in relation to the mock-treated controls.

Since the large amounts of IFN-γ were inversely correlated with the antibody titers in the PMNd-*Br* mice, we injected nonsterilizing amounts of anti-*Brucella* antibodies at different infection times. Anti-*Brucella* serum given 1 day before infection completely abrogated the IFN-γ response in mice and lowered the bacterial loads (Fig. 9), regardless of the presence or absence of PMNs. However, if the same antibody regime was given after 6 days of infection, the levels of IFN-γ remained unchanged (Fig. 9B). Moreover, after treatment with the corresponding antibodies at 6, 9, and 12 days after infection (see Fig. S1), the levels of IFN-γ were not significantly different at 14 days of infection (Fig. 9C), although the bacterial loads were still lower than in the mock-treated controls (Fig. 9E).

### Anti-*Brucella* antibodies abrogate IL-6, IL-10, and IL-12 cytokines in neutropenic mice.

**FIG 10:**
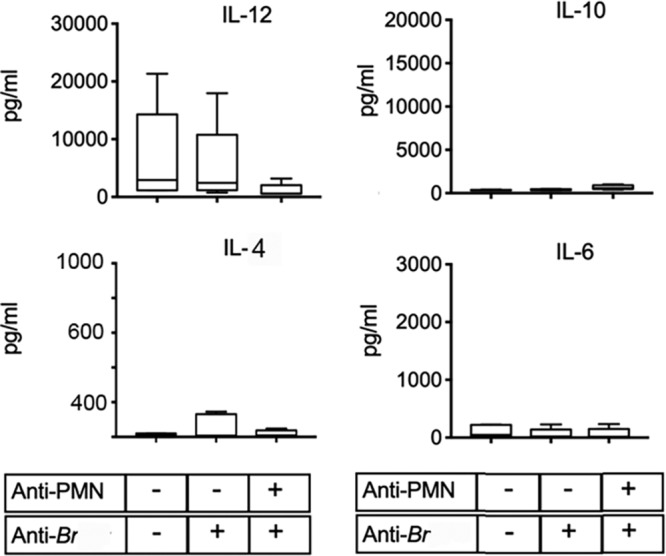
Anti-*Brucella* antibodies dampen proinflammatory cytokines in neutropenic mice at acute stages of infection. C57BL/6 mice were i.p. injected (+) or not (−) either with immune mouse sera against *Brucella* or with a mixture of immune mouse sera against *Brucella* and anti-PMN (RB6-8C5) at 6, 9, and 12 days after infection, and the various proinflammatory cytokines were measured in the sera of the mice by ELISA after 14 days of infection. Median values are indicated by the horizontal lines within the bars. In comparison with cytokine levels in neutropenic infected mice shown in Fig. 4 (far right column in each graph), the levels of IL-12, IL-10, and IL-6 were significantly lower (*P* < 0.01) in neutropenic mice treated with anti-*Brucella* antibodies.

Since IL-6, IL-10, and IL-12 cytokines were considerably elevated in PMNd-*Br* mice at later stages of acute infection (day 14 of infection; day 9 postdepletion) (Fig. 4), we explored the effects of anti-*Brucella* antibodies at these times in PMNd-*Br* mice. In contrast to IFN-γ, anti-*Brucella* antibodies dampened the levels of IL-6, IL-10, and IL-12 in the PMNd-*Br* mice (Fig. 10; compare with Fig. 4). Other cytokines, such as IL-4, tumor necrosis factor alpha (TNF-α), and IL-1β, remained close to background levels at all times and in all experiments (Fig. 4 and 10).

## DISCUSSION

We have shown that PMN removal before the development of adaptive immunity promotes the elimination of B. abortus from target organs at the onset of infection (4). This phenomenon is linked to the efficient recruitment of macrophages and dendritic cells, stronger activation of CD4^+^ and CD8^+^ T lymphocytes, and the concomitant increase of IFN-γ (4). Here, we have complemented these findings and demonstrated that the absence of PMNs, after adaptive immunity has fully developed, also favors the efficient elimination of B. abortus in mice.

These results seem counterintuitive, mainly when they are compared to the positive role that PMNs play in controlling other bacterial infections, such as those by *Salmonella*, *Yersinia, Legionella*, and *Listeria* organisms (7, 8, 11). In the case of *Brucella* sp. infections, the primary microbicidal function of PMNs is not achieved. Rather, *Brucella* organisms induce the premature death of PMNs in a nonphlogistic manner (19) and dampen the regulatory influence that PMNs have on adaptive immunity at different stages of an infection.

It is known that M1 macrophages are the first line of defense against intracellular pathogens, including *Brucella* organisms (32, 33). The higher production of IFN-γ is correlated with the activation of these cells, the resolution of inflammation, and the efficient elimination of bacteria in PMNd-*Br* mice. Under the influence of IFN-γ, M1 macrophages differentiate, increase their microbicidal activity, and amplify Th1 polarization of CD4^+^ lymphocytes by IL-12 production (33, 34).

Higher secretion of IFN-γ was a common feature in both the PMNd-*Br* mice at the onset of infection (4) and PMNd-*Br* mice after adaptive immunity had emerged. In spite of this, some significant differences were observed. For instance, the levels of IFN-γ produced at the onset of infection were lower (4) than those recorded once adaptive immunity had developed. While in the former case the levels of IFN-γ were not associated with sickness, in the latter case weight loss and cachexia were observed in the neutropenic infected mice. This was an unexpected clinical feature. Indeed, *Brucella*-infected mice seldom show sickness during the early days of infection (23). It seemed, therefore, that the very high levels of IFN-γ (close to 7,500 pg/ml) and the subsequent activation of the immune system were not without a price (35).

It has been shown that IL-12 is an essential cytokine to retain a Th1 response in brucellosis (36). The higher levels of IL-12 at later times of acute infection in the PMNd-*Br* mice revealed no shift toward a Th2 response. Moreover, cytokine IL-4 always remained close to background levels. The negligible amount of IL-4 in the sera and spleen cells of infected mice during brucellosis is a well-known feature and delineates the Th1 predominant immune response (15, 23). It is also known that removal of IL-4 depresses anti-*Brucella* antibody response, indirectly favoring the Th1 response (37).

The rise of IL-10 and IL-6 in the PMN-depleted infected mice at later times of infection correlated with the decreasing levels of IFN-γ. The lack of IL-10 has been related to lower B. abortus survival at early stages of infection and linked to the regulation of IFN-γ at later stages (31, 37). Likewise, IL-6 limits the recruitment of innate immune cells and therefore represents a critical element in the regulation of inflammation (38). A rise in IL-6 has also been observed at the onset of infection in PMNd-*Br* mice, including the Genista strain (4).

It is worth noting that anti-*Brucella* antibodies dampened IFN-γ only when given before infection. This seems to be related to the fast removal of bacteria, which hampers the development of adaptive immunity, a trend observed previously with other bacteria (39, 40). However, after the initiation of adaptive immunity, anti-*Brucella* antibodies did not influence the levels of IFN-γ, regardless of the presence or absence of PMNs or the number of bacteria. This agrees with previous data showing that the levels of IFN-γ generated are independent of the bacterial load in B. abortus-infected mice (4). In contrast, anti-*Brucella* antibodies dampen IL-6, IL-10, and IL-12 in neutropenic mice at later stages of acute infection (compare Fig. 4 with Fig. 10), a fact that correlates with the lower numbers of bacteria in the treated mice.

It was clear that the efficient elimination of bacteria in the neutropenic mice was not linked to the rise of antibodies or to increased levels of specific immunoglobulin isotypes against *Brucella* antigens. On the contrary, lower antibody titers were observed after 3 and 4 weeks of infection. This may be the result of a stronger cellular immunity, promoted by the high levels of IFN-γ during the acute phase of infection. The fact that IFN-γ exerts a regulatory influence on the production of immunoglobulin isotypes against *Brucella* antigens supports this (30). In addition, mice devoid of B cells (and thus deprived of antibodies) eliminate B. abortus more efficiently, which is linked to higher levels of IFN-γ and to a stronger cellular Th1 response (41). Although the lower bacterial loads could have had some influence on the antibody titers, this seems unlikely. It has been shown that once antibodies are produced, they remain at the same high levels, regardless of the number of *Brucella* organisms present in the target organs (42). Likewise, an increase in bacterial loads was recorded in the PMNd-*Br* mice on the first day after PMN removal (Fig. 1 and 2).

The ability of *Brucella* organisms to produce chronic infection is linked to their long persistence in the BM (17, 43). In humans and mice, colonization of the BM by *Brucella* organisms causes neutropenia, thrombocytopenia, anemia, pancytopenia, and other pathological signs (17, 43, 44). The bacterium resides within BM monocytes, PMNs, and, to a lesser extent, granulocyte-monocyte progenitors (17). Therefore, the abrogation of the B. abortus infection in the BM of PMNd-*Br* mice was intriguing, considering the significant number of PMNs remaining in the BM after repeated injections of anti-PMN (see Table S1). Whether anti-PMN antibodies remove mostly mature and functional PMNs from the BM remains to be studied.

The precise routes by which PMNs regulate other cells of the immune system remain elusive, and those proposed for other pathogens do not match our observations (45, 46). For instance, in a murine model of Legionella pneumophila infection, PMN depletion led to more Th2 skewing and more disease (8). This is striking, since in both bacterial diseases IFN-γ plays a central role (28, 29, 47), and the pathogenic mechanisms and intracellular life cycles of the two bacteria display some resemblances (48). Other regulatory mechanisms, such as direct contact between PMNs and lymphocytes, macrophages/monocytes, and dendritic cells, have been discussed (4). Regulation through PMN cytokines seems unlikely, since the amounts of proinflammatory cytokines released by B. abortus-infected PMNs are negligible (19).

One alternative mechanism that explains the phenomenon observed here is related to the “Trojan horse” hypothesis (19, 24). This mechanism proposes that prematurely dying *Brucella*-infected PMNs displaying “eat me” signals are readily phagocytized by cells of the mononuclear phagocytic system in a nonphlogistic manner (19). This opens a window for the intracellular trafficking of brucellae to the endoplasmic reticulum and eventual replication in these phagocytic cells. This delays the activation of the adaptive immune system, allowing the stealthy organism to establish a long-lasting infection (11, 15, 24). In the absence of PMNs, this mechanism is shattered, allowing mononuclear phagocytic cells to interact directly with the bacterium in a proinflammatory manner. This allows strong activation of the immune system, reflected by increased release of IFN-γ by CD4^+^ and CD8^+^ cells and polarization of macrophages toward M1, which is central to combating intracellular parasites. This proposal fits the Occam’s razor principle of parsimony, previous experimental data, and the results presented here.

## MATERIALS AND METHODS

### Ethics.

Experimentation with mice was conducted following the guidelines of the Comité Institucional para el Cuido y Uso de los Animales of the Universidad de Costa Rica (CICUA-019-16) and in agreement with the corresponding law (Ley de Bienestar de los Animales de Costa Rica; law 9458 on animal welfare). Mice were housed in the animal facility of the Veterinary Medicine School of the National University of Costa Rica. The mice were kept in cages with food and water *ad libitum* under biosafety conditions.

### Generation of neutropenic mice.

Inbred C57BL/6 mice (18 to 21 g) were used in the experiments. Neutropenic mice were generated as previously described (4, 15). Briefly, mice were depleted of PMNs by means of intraperitoneal (i.p.) injection of 100 μg of rat anti-mouse Ly-6G/Ly-6C (Gr-1) (clone RB6-8C5; Bio X Cell) or 500 μg of anti-mouse Ly6G (clone 1A8; BD Biosciences) in 0.1 ml phosphate-buffered saline (PBS). PMN depletion was confirmed by the absence of CD11b^+^ Ly6G^+^ cells by flow cytometry and microscopic examination of blood, spleen, lymph nodes, and BM (4) (see Table S1). A single i.p. injection of anti-PMN antibody resulted in the depletion of PMNs from blood, spleen, and lymph nodes for at least 3 days (see Table S1). PMN depletion in the BM was achieved only to 25 to 30% (see Table S1). Differences in depletion were observed between RB6-8C5 and 1A8 antibodies. In order to maintain the neutropenic stage, mice were injected with the indicated antibody every 3 days according to the following protocols: (i) depletion at the onset of innate immunity, (ii) depletion at the onset of adaptive immunity, and (iii) depletion at the acute phase of infection (see Fig. S1). In all experiments, nonimmune rat IgG was used and administered at the same concentrations and by the same route as the anti-PMN antibodies (mock-treated controls). After 8 days from the first anti-PMN injection, the mice developed antibodies against the anti-PMN antibody (4). A detailed time course protocol and kinetics for the RB6-8C5-PMN depletion after B. abortus infection has been reported previously (4).

### Brucella abortus infection.

Mock-treated and PMN-depleted mice were i.p. infected with 0.1 ml of PBS containing 10^6^ CFU of virulent B. abortus 2308W (49) as described previously (4). Bacterial colonization was determined in spleens and BM of mice collected at the indicated times, following previously published protocols (4, 17, 50). Serial dilutions of infected macerated tissues were plated on Trypticase soy agar and incubated at 37°C for 72 h in the presence 5% CO_2_, and bacterial CFU were determined (50). Spleens from the mice were processed for histopathological studies as described previously (51). Blinded evaluation of histopathology slides was performed. The inflammatory stage was evaluated using a semiquantitative scoring system (31).

### Antibody and cytokine determination.

Murine hyperimmune serum production against *Brucella* antigens and IgG purification were carried out following previously published protocols (21). PMN-depleted mice and the mock-treated control mice were bled at different times, serum was separated from cells, and antibody titration was carried out in 96-well round-bottom plastic plates as described previously (21). After titration, immune sera were stored at −20°C in aliquots. Western blotting revealed that most antibody recognition was directed against *Br*-LPS.

For isotype antibody determination against *Br*-LPS, enzyme-linked immunosorbent assays (ELISAs) were performed on 96-well plates (Nunc) as previously described (52). Briefly, the 96-well plates were coated with 0.1 ml of 10-μg/ml *Br*-LPS. Mouse serum was diluted 1:200 in blocking buffer (PBS with 0.4% bovine serum albumin [BSA] and 0.05% Tween 20) and then incubated on plates for 1 h at 37°C, followed by extensive washing (PBS with 0.05% Tween 20). Secondary horseradish peroxidase (HRP) antibody conjugates against mouse IgG, IgM, IgG1, IgG2a, IgG2b, and IgA (all from Sigma-Aldrich) at the adjusted dilution in the blocking buffer were used for immunoglobulin isotyping. After washing the plates, the reaction was developed with HRP substrate (Sigma-Aldrich), and the optical density was measured at 450 nm. Serial dilutions of the murine hyperimmune serum (positive-control serum) and the respective conjugates were performed in order to establish the optimal cutoff value for each conjugate in comparison to sera from uninfected mice. The negative-control serum optical density for each conjugate was adjusted to 0.110 ± 0.025 nm, while the positive-control serum optical density was adjusted to 1.200 ± 0.150 nm. The cutoff value was estimated at 0.200 nm.

The levels of IL-1β, IL-2, IL-4, IL-6, IL-10, IL-12, IFN-γ, and TNF-α cytokines were measured in sera by ELISA (eBioscience), according to the manufacturer’s specifications.

### Flow cytometry.

Flow cytometry was carried out as previously described (4). Phycoerythrin (PE) anti-CD11b (M1/70), Alexa Fluor 488 anti-Ly6C (AL-21), and PE cyanine 5.5 anti-Ly6G (1A8) antibodies were purchased from BD Biosciences, and 1A8 and RB6-8C5 neutralizing antibodies were from Bio X Cell. Blood, spleen, and bone marrow cells were prepared as described previously (4, 17). Popliteal, inguinal, and mesenteric lymph nodes were prepared as described previously (53) and processed for flow cytometry as described previously (4). Intracellular staining was performed with allophycocyanin (APC) anti-iNOS (clone CXNFT) and PE anti-IL-6 (clone MP5-20F3) with the respective isotype controls, all from Invitrogen. Before staining with different antibody mixtures, cells were preincubated on ice for at least 10 min with the anti-mouse CD16/CD32 (clone 2.4G2) monoclonal antibody to block Fc receptors (BD Biosciences). Multiparameter fluorescence-activated cell sorter (FACS) analysis was performed, using a Guava easyCyte flow cytometer (Millipore). The FACS data were analyzed using Flow Jo software, version 10.4. For each experiment, control mice were included to define the proper gates. Blood was stained directly with the antibodies and lysed with BD FACS lysing buffer (BD Biosciences). If the mice had been previously treated with PMN-depleting antibodies, the blood samples were washed thoroughly (four times) with PBS to remove anti-Ly6G from the sera before the staining process. All the samples were washed and resuspended in PBS prior to acquisition.

### Statistics.

The data were processed in Microsoft Office Excel. To determine statistical significance, comparison of two samples was performed by a Mann-Whitney test, and multiple comparisons were established by a Kruskal-Wallis test using the GraphPad software package (version 7.0; GraphPad, La Jolla, CA, USA). For antibody isotype comparison, values were normalized by adjusting the measurements of the different scales to a notionally common scale. For all tests, *P* values of <0.05 and <0.01 were considered statistically significant.

## Supplementary Material

Supplemental file 1

Supplemental file 2

Supplemental file 3

Supplemental file 4

Supplemental file 5

Supplemental file 6
