# Supplementary material for: Neutrophils Dampen Adaptive Immunity in Brucellosis
Source: Infect Immun. 2019 Apr 23;87(5):e00118-19. doi: 10.1128/IAI.00118-19 (PMC6479033; doi:10.1128/IAI.00118-19)
Supplement: Supplemental file 1 [file IAI.00118-19-s0001.pdf]

SUPPLEMENTARY FIGURE LEGENDS

Figure S1. Experimental design. Top black arrows indicate the days at which a group of mice was treated with anti-RB6-8C5 or 1A8 antibodies for PMN depletion, or with non-immune rat IgG as mock-control. Bottom black arrows indicate the days of i. p. infection with 0.1 mL containing 10^6 CFUs *B. abortus* 2308W, determination of CFU counts, histopathology, and cytokines in the various groups of mice. PMNs basal level over time after treatment with anti-RB6-8C5 is shown with a blue dashed line at the indicated times. The red arrows indicate the proportion of PMNs at the indicated times.

Figure S2. PMN depletion with 1A8 antibody during the acute infection period promotes *Brucella* removal. C57BL/6 mice were i. p. infected with 0.1 mL containing10^6 CFUs of *B. abortus* 2308W. After 15 days of infection, one group of mice was depleted of PMNs by means of i. p. injection of 1A8 anti-PMN. (A) Then at the indicated times CFU/spleen and spleen weights were determined. Each symbol represents one animal, and the lines represent the median for each group. (B) Rate of change in CFU/spleen (Δ CFU/spleen) and CFU/spleen weight (Δ CFU/g of spleen) were calculated over time using the following equations: ΔCFU/spleen=mean CFUs 30 days/CFUs 6 day ± SD; ΔCFU/g of mean spleen= CFU/ g of spleen 30 days/16 day ± SD. Bars represent standard deviation. Values of $p<0.01$ (**) in relation to the mock-controls are indicated below the bars.

Figure S3. PMN depletion with 1A8 antibody during the acute infection period promotes increased levels of IFN-γ. Mice were i. p. infected with 10^6 CFUs of *B. abortus* 2308W. After five days post-infection, one group of mice was depleted of PMNs by means of i. p. injection of 1A8 anti-PMN. Then at the indicated times IFN-γ levels were determined by ELISA in the sera of all mice at 6 and 14 days post-infection, respectively (1 and 9 days post-depletion). Horizontal lines represent the median for each group. Values of $p<0.05$ (*) indicated below the bars, are in relation to the values of the mock-control mice.

Fig S4. The antibody responses against *B. abortus* antigens is depressed in mice depleted of PMN with 1A8 anti-PMN at the acute stages of infection. C57BL/6 mice were i. p. infected with 0.1 mL containing 10^6 CFUs of *B. abortus* and at 5 days of infection one group of mice was depleted of PMNs by means of i. p. injection of 1A8 anti-PMN. (A) Agglutination titer against *Brucella* cells. (B) Isotype antibody responses against *Br*-LPS. Each symbol represents one animal. Blue dashed lines show the average of the mock-controls and the grey areas represent the standard deviation of the mock-controls.
