# Supplementary figures and images for: Neutrophils Dampen Adaptive Immunity in Brucellosis
Source: Infect Immun. 2019 Apr 23;87(5):e00118-19. doi: 10.1128/IAI.00118-19 (PMC6479033; doi:10.1128/IAI.00118-19)

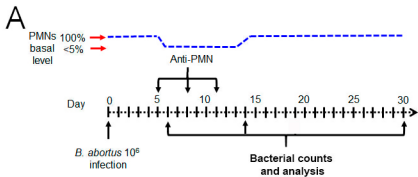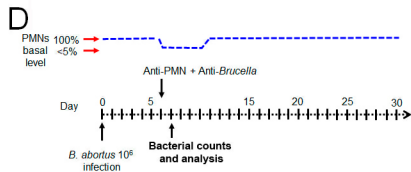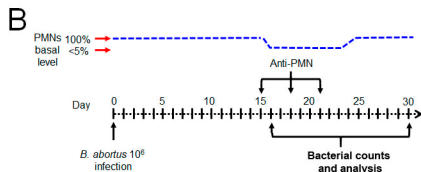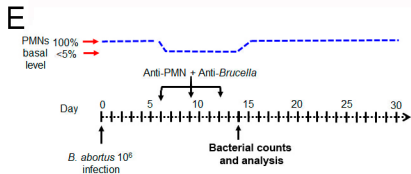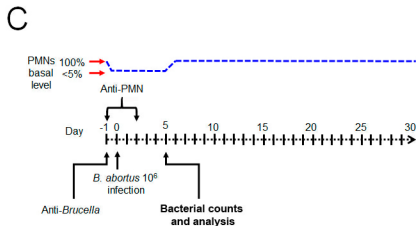

Supplement: Supplemental file 2 [file IAI.00118-19-s0002.pdf]

**A**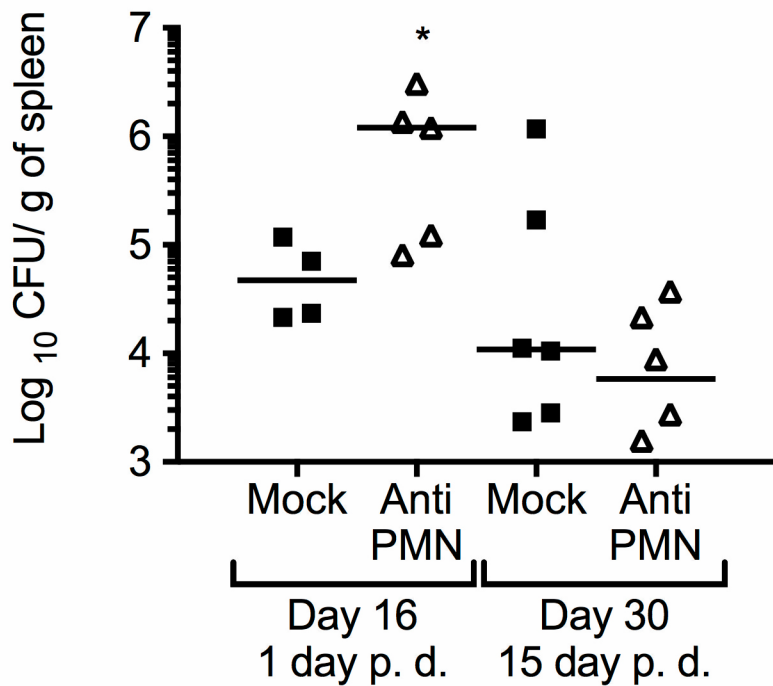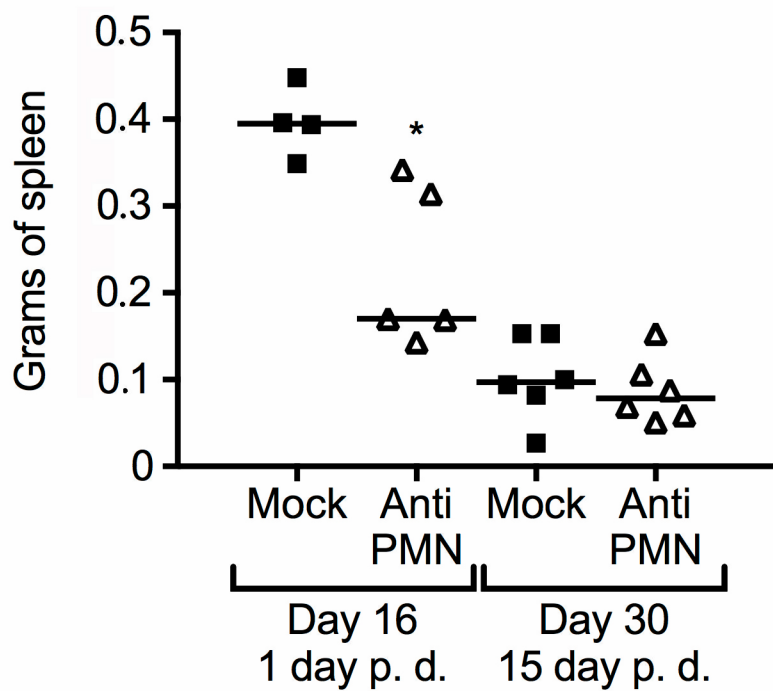**B**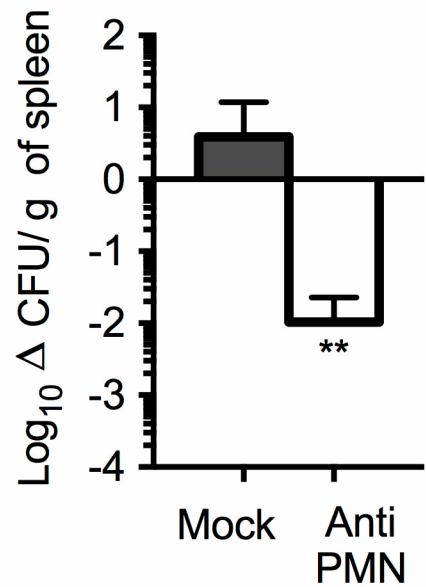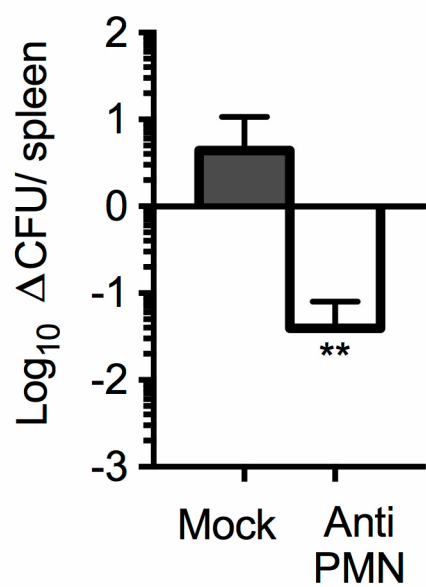

Supplement: Supplemental file 3 [file IAI.00118-19-s0003.pdf]

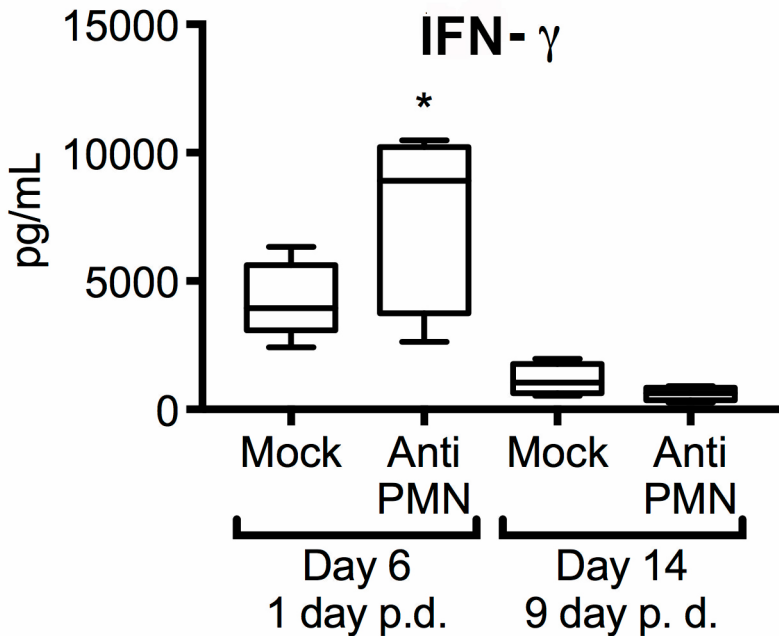

Supplement: Supplemental file 4 [file IAI.00118-19-s0004.pdf]

**A**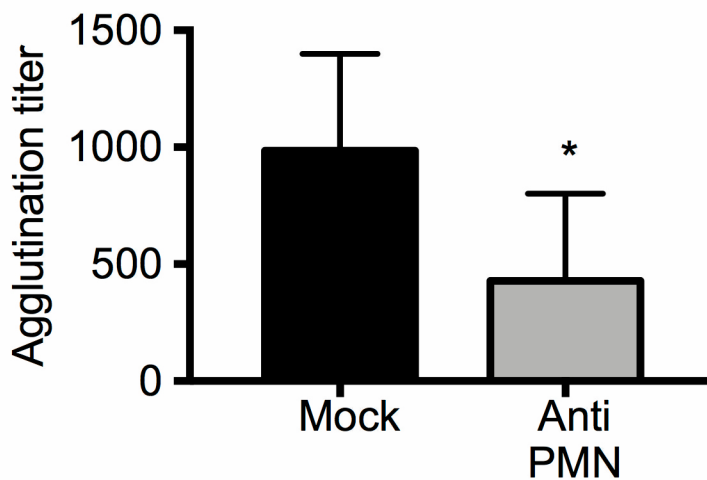**B**

30 days  
(25 days p.d.)

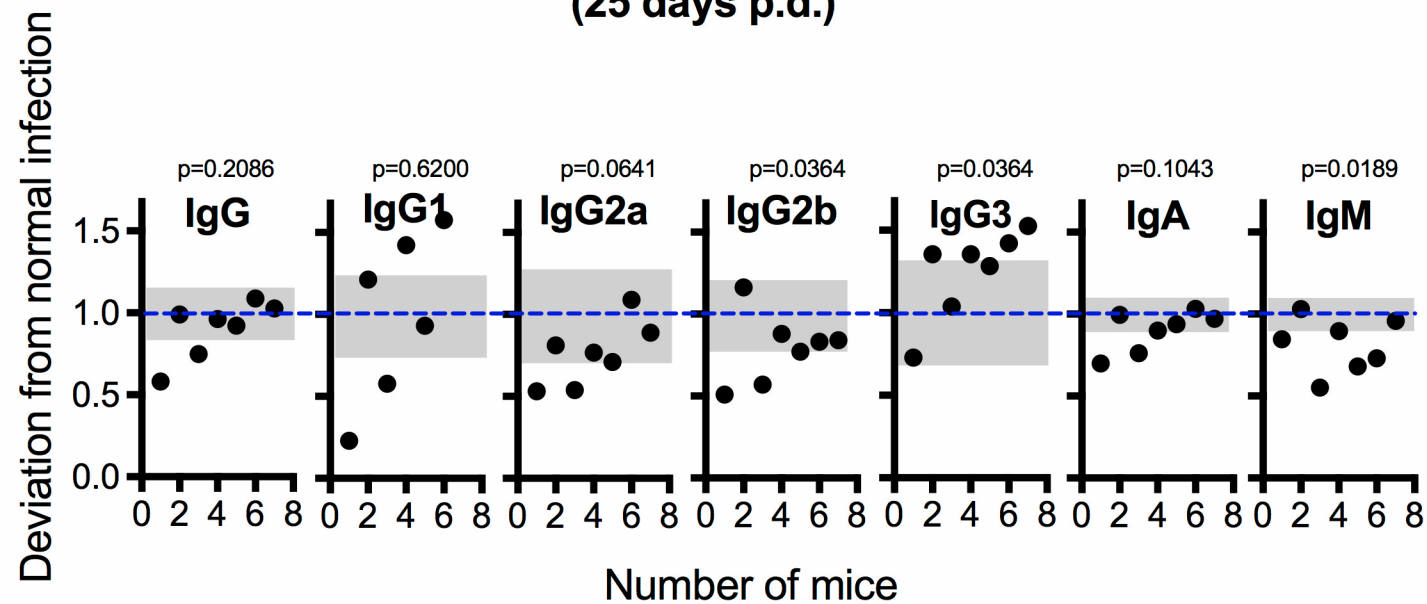

Supplement: Supplemental file 6 [file IAI.00118-19-s0006.pdf]
