# Supplementary material for: Neutrophils Dampen Adaptive Immunity in Brucellosis
Source: Infect Immun. 2019 Apr 23;87(5):e00118-19. doi: 10.1128/IAI.00118-19 (PMC6479033; doi:10.1128/IAI.00118-19)
Supplement: Supplemental file 5 [file IAI.00118-19-s0005.pdf]

Table S1. Confirmation on the presence of PMNs and monocytes in tissues of *B. abortus* infected mice, untreated and treated with anti-PMN antibodies

Tissue	Time of infection/depletion	Cell type	Treatment		
			Mock	Anti-PMN 1A8	Anti-PMN RB6-8C5
Blood	Day 6 of infection (1-6 days post-depletion)	PMNs	21.3±7.9	3±2	0.5±0.3
		Monocytes	32.9±12.1	30.5±2.2	8.35±8
	Day 14 of infection (9 days post-depletion)	PMNs	28.7±3.5	ND	39.28±18.3
		Monocytes	25.4±3.7	ND	25.4±4.96
Bone Marrow	Day 6 of infection (1-6 days post-depletion)	PMNs	34.4±2.4	ND	25.1±3.4
Spleen	Day 6 of infection (1-6 days post- depletion)	PMNs	1.8±0.8	ND	0.3±0.6
Lymph nodes	Day 6 of infection (1-6 days post- depletion)	PMNs	<0.5	NS	<0.5
